# Characterization of the biochemical properties of *Campylobacter jejuni* RNase III

**DOI:** 10.1042/BSR20130090

**Published:** 2013-11-25

**Authors:** Nabila Haddad, Margarida Saramago, Rute G. Matos, Hervé Prévost, Cecília M. Arraiano

**Affiliations:** *LUNAM Université, Oniris, UMR1014, Secalim, Nantes, F-44307, France; †INRA, Nantes, F-44307, France; ‡Instituto de Tecnologia Química e Biológica, Universidade Nova de Lisboa, Avenida da República, 2780-157 Oeiras, Portugal

**Keywords:** *Campylobacter jejuni*, catalytic activity, manganese, ribonuclease, RNase III, *Cj*-RNase III, *C. jejuni* RNase III, cpm, counts/min, dsRBD, dsRNA binding domain, DSS, disuccinimidyl suberate, DTT, dithiothreitol, *Ec*-RNase III, *E. coli* RNase III, LB, Luria–Bertani, PNPase, polynucleotide phosphorylase, RNase III, ribonuclease III, wt, wild-type

## Abstract

*Campylobacter jejuni* is a foodborne bacterial pathogen, which is now considered as a leading cause of human bacterial gastroenteritis. The information regarding ribonucleases in *C. jejuni* is very scarce but there are hints that they can be instrumental in virulence mechanisms. Namely, PNPase (polynucleotide phosphorylase) was shown to allow survival of *C. jejuni* in refrigerated conditions, to facilitate bacterial swimming, cell adhesion, colonization and invasion. In several microorganisms PNPase synthesis is auto-controlled in an RNase III (ribonuclease III)-dependent mechanism. Thereby, we have cloned, overexpressed, purified and characterized *Cj*-RNase III (*C. jejuni* RNase III). We have demonstrated that *Cj*-RNase III is able to complement an *Escherichia coli rnc-*deficient strain in 30S rRNA processing and PNPase regulation. *Cj*-RNase III was shown to be active in an unexpectedly large range of conditions, and Mn^2+^ seems to be its preferred co-factor, contrarily to what was described for other RNase III orthologues. The results lead us to speculate that *Cj*-RNase III may have an important role under a Mn^2+^-rich environment. Mutational analysis strengthened the function of some residues in the catalytic mechanism of action of RNase III, which was shown to be conserved.

## INTRODUCTION

Bacteria are able to rapidly adjust their physiology in order to survive in response to environmental demands. This adaptation is accompanied by a fast adjustment of the RNA levels. The cellular concentration of a given RNA is the result of the balance between its synthesis and degradation. RNA degradation involves the concerted action of ribonucleases. Changes in RNA turnover facilitate stress responses, growth phase transitions and production of virulence factors [1,2].

RNase III (ribonuclease III) family of enzymes is a highly conserved group of ds (double-stranded) RNA-specific endoribonucleases widely distributed among prokaryotic and eukaryotic organisms. In bacteria, RNase III has the ability to regulate its own synthesis with a specific cleavage near the 5′end of its mRNA. Moreover, it is also responsible for rRNA operon maturation, processing of cellular, phage and plasmid RNAs, and decay of sRNA/mRNA complexes upon translational silencing [1,3,4].

**Figure 1:**
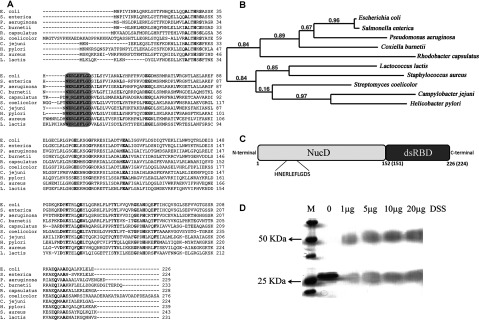
*C. jejuni* RNase III protein features (**A**) Sequence alignment of RNase III from *E. coli* (Uniprot ID: P0A7Y0), *S. enterica* (Uniprot ID: E7V351), *P. aeruginosa* (Uniprot ID: B7UYX2), *C. burnetii* (Uniprot ID: P51837), *R. capsulatus* (Uniprot ID: Q52698), *S. coelicolor* (Uniprot ID: Q9ZBQ7)*, C. jejuni* (NCBI Reference Sequence: YP_001001278), *H. pylori* (Uniprot ID: P56118)*, S. aureus* (Uniprot ID: P66668) *and L. lactis* (Uniprot ID: Q9CHD0). Fully conserved residues are in bold, and the signature sequence of these proteins is highlighted. (**B**) Phylogenetic tree of RNase III from the species considered in (a). (**C**) Schematic representation of the domain organization of RNase III, showing the catalytic domain (NucD) (residues 1 to 151 in *E. coli* protein and 1 to 150 in *C. jejuni* protein) and the dsRNA binding domain (residues 152–226 in *E. coli* protein and 151–224 in *C. jejuni* protein). The signature sequence present in the catalytic domain is emphasized. (**D**) Cross-linking of RNase III from *C. jejuni* using DSS. Approximately 0.5 μg of protein was incubated with increasing concentrations of DSS as indicated in the figure. Proteins were visualized by Coomassie Brilliant Blue staining.

Bacterial RNase III is the simplest member of the family, containing an endonuclease domain (NucD), characterized by the presence of a set of highly conserved carboxylic acid residues essential for catalytic activity, and a dsRBD (dsRNA binding domain) (Figure 1C). In *Escherichia coli*, this enzyme is encoded by the *rnc* gene and it is active as a 52 kDa homodimer [3].

Important insights on structural features and mechanism of action of RNase III have been provided by crystallographic studies. Structural analysis of *Aquifex aeolicus* RNase III revealed a symmetric positioning of the two catalytic domains that form the dimer through hydrophobic interactions, and demonstrated a positional mobility of the dsRBD [5]. A divalent metal ion is required for RNase III activity, with magnesium (Mg^2+^) as the preferred co-factor. This homodimer uses a two metal mechanism of catalysis, with each active site containing two divalent cations during substrate hydrolysis [6]. The structure of *Thermotoga maritime* together with the one from *A. aeolicus* provided the basis for a proposed pathway of dsRNA recognition and cleavage by the bacterial RNase III members [7]. Accordingly, RNase III can affect gene expression in two different ways. When the dsRNA is not bound to the catalytic valley, RNase III binds it without cleaving [8]. In this form, RNase III can affect RNA structures and modulates gene expression [9,10]. In addition, it can also function as a dsRNA-processing enzyme, cleaving both natural and synthetic dsRNA [5]. The RNase III cleavage originates 5′ phosphate and 3′ hydroxyl termini with a two-base overhang at the 3′end [5]. In eukaryotes, homologues of this enzyme have a key role in RNA interference phenomenon that starts with a dsRNA cleavage [3].

*Campylobacter jejuni* is a Gram-negative microorganism, which remains as one of the major causes of human gastroenteritis worldwide [11]. The main reservoir of *C. jejuni* is the guts of avian species with up to 10^9^ CFU (colony forming units)/g in faeces [12]. Owing to the spillage of intestinal content that contains a large number of the pathogen, *C. jejuni* can contaminate cooling water, knives and poultry meat during slaughter. Thus, the consumption of contaminated poultry is the major cause of campylobacteriosis; however, a diverse range of environmental sources are also all recognized causes of infection [13]. Despite the specific microaerobic growth requirements, *C. jejuni* is ubiquitous in the aerobic environment and is capable to face different stresses during transmission, including temperature changes, starvation, hypo- and hyper-osmotic stress, and desiccation. In addition, during human infection, *C. jejuni* has to withstand a further range of stresses, including changes in pH and the host innate immune response [14,15]. Although major advances have been made in the understanding of *C. jejuni* pathobiology and physiology, many unanswered questions remain. A more complete understanding of the regulation of *C. jejuni* response mechanisms is required to facilitate appropriate intervention strategies in order to reduce *C. jejuni*-associated diseases [16].

It was demonstrated by Haddad et al. [17,18] that the exoribonuclease PNPase (polynucleotide phosphorylase) is involved in low-temperature survival of *C. jejuni*, and that the lack of PNPase induces swimming limitation, chick colonization delay and decrease of cell adhesion/invasion ability. However, little is known regarding RNases in this pathogen. In *E. coli*, PNPase synthesis is autocontrolled at a post-transcriptional level in an RNase III-dependent mechanism [19,20]. The functional and evolutionary conservation of the RNase III family in bacteria and higher organisms is indicative of their biological relevance.

We present here the relevant information regarding functionality and biochemical properties of *Cj*-RNase III (*C. jejuni* RNase III). We show that this endoribonuclease is able to replace *Ec*-RNase III (*E. coli* RNase III) in the 30S processing and in the regulation of PNPase levels. We also demonstrate the dependence of *Cj*-RNase III catalytic activity on divalent metal ions, and its ability to cleave at different temperatures and pH values that mimic different physiological environments that *C. jejuni* may face. Finally, a biochemical analysis was assessed on *Cj*-RNase III mutants with substitutions on two conserved acidic residues in the nuclease domain.

## EXPERIMENTAL

### Bacterial strains and culture conditions

The strains used in this study are listed in Table 1. *C. jejuni* 81-176 was routinely cultured on Karmali agar plates (Oxoid) or in BHI (brain heart infusion) broth (Merck). Cultures on plates and broth were incubated at 42°C for 48 and 24h, respectively, under a microaerophilic atmosphere in jars flushed with a gas mixture of 10% (v/v) CO_2_, 5% (v/v) O_2_ and 85% (v/v) N_2_. *E. coli* strains were cultivated overnight at 37°C in the LB (Luria–Bertani) medium (Sigma). The growth medium was supplemented with ampicillin (100 μg/ml) when appropriate.

**Table 1:** Strains used in this study

Strain	Antibiotic resistance	Reference	Genetic
*E. coli* DH5α	–	Invitrogen	*rec*A1 *end*A1
BL21(DE3) *rec*A*rnc*105	Chloramphenicol	[22]	*rec*A::Tn9Δ*rnc*105
*E. coli* MG1693	–	[47]	thyA715
*E. coli* SK7622	Kanamycin	[30]	thyA715 Δ*rnc*
*C. jejuni* 81-176	–	[48]	–

#### Construction of the plasmids expressing RNase III from *C. jejuni*

The *C. jejuni* 81–176 RNase III gene (*rnc*) (GenBank accession no. YP_001001278) was amplified from genomic DNA using the primers MO608 (5′-AATTCATATGCAAGGGAAAATGATGAAAAA -3′) and MO612 (5′- GTAGGATCCAATTTCAATCTCGTACCAAAGGTAT -3′) (Table 3) (*Nde*I and *Bam*HI sites, respectively, are underlined). For *rnc* amplification by PCR, 1 unit of Phusion High-Fidelity DNA Polymerase (BioLabs) was used. The purified PCR product (734 pb) was cloned into the pGEM-T plasmid (Promega) to generate pGEMT-*rnc*. To overexpress the *Cj*-RNase III protein, the *rnc* gene double digested with *Nde*I and *Bam*HI was subcloned from pGEMT-*rnc* into the pET19b expression vector previously digested with the same enzymes (Novagen). Plasmids and primers used are presented in Tables 2 and 3, respectively. The resultant pET19b-*rnc* was confirmed by DNA sequencing (Beckman Coulter Genomics).

**Table 2:** Plasmids used in this study

Plasmid	Antibiotic resistance	Reference	Comments
pGEM-T	Ampicillin	Promega	Commercial vector
pET19b	Ampicillin	Novagen	Commercial expression vector
pGEMT:*rnc*	Ampicillin	This study	Encodes Cj-RNC
pET19b-*rnc*	Ampicillin	This study	Encodes his-Cj-RNC
pET19b-*rnc*_D44A	Ampicillin	This study	Encodes his-Cj-RNC_D44A mutant
pET19b-*rnc*_E116Q	Ampicillin	This study	Encodes his-Cj-RNC_E116Q mutant
pET19b-*rnc*_E116D	Ampicillin	This study	Encodes his-Cj-RNC_E116D mutant

**Table 3:** Primers used in this study

Primers	Sequence (5′-3′)	Restriction site
MO608	AATTCATATGCAAGGGAAAATGATGAAAAA	*Nde*I
MO612	GTAGGATCCAATTTCAATCTCGTACCAAAGGTAT	*Bam*HI
Cjej_9	CATCACAGCAGCGGC	
Cjej_10	GCAGCCGGATCCTAT	
D44A_1	GGTgcaGCTGTGCTTG	Introduction of D44A mutation and *Pvu*II site
D44A_2	CAAGCACAGCtgcACC	
E116Q_1	CAGAcGCGTTAGAtGCCAT	Introduction of E116Q mutation and *Mlu*I site
E116Q_2	ATGGCaTCTAACGCgTCTG	
E116D_1	CAGAcGCGTTAcAAGCCAT	Introduction of E116D mutation and *Mlu*I site
E116D_2	ATGGCTTgTAACGCgTCTG	

#### Construction of RNase III mutants by overlapping PCR

The point mutations D44A, E116Q and E116D were introduced into pET19b-*rnc* by overlapping PCR [21]. The primers used are listed in Table 3 and base changes are indicated in small letters. All mutant constructs were confirmed by DNA sequencing at STAB Vida, Portugal.

#### Overexpression and purification of recombinant wt (wild-type) RNase III and mutants from *C. jejuni*

The plasmids harbouring the histidine-tagged *Cj*-RNase III wt protein (pET19b-*rnc*) and the mutants (pET19b-*rnc*_D44A, pET19b-*rnc*_E116Q, pET19b-*rnc*_E116D) were transformed into *E. coli* BL21(DE3) *rec*A*rnc*105 strain ([22], kindly provided by Professor A. Nicholson to allow the expression of the recombinant proteins. This derivative strain of BL21(DE3), carrying an RNase III mutation, was used since it blocks the regulation of *Cj*-RNase III by the endogenous *E. coli* homologue, resulting in a higher yield of the enzyme upon overexpression. Cells were grown at 37°C in 100 ml LB medium supplemented with 100 μg/ml ampicillin to an optical density of 0.5 at 600 nm. At this point, protein expression was induced by addition of 0.5 mM IPTG and left to grow for 2 h. Cells were pelleted by centrifugation and stored at −80°C. Purification was performed by histidine affinity chromatography using HiTrap Chelating HP columns (GE Healthcare) and AKTA FPLC system (GE Healthcare) following a protocol previously described [4]. The purity of the proteins was verified in a SDS/12%PAGE gel followed by Coomassie blue staining. Proteins were quantified using the Bradford Method [23] and 50% (v/v) glycerol was added to the final fractions prior storage at −20°C.

### Cross-linking of *Cj*-RNase III wt and mutants

In order to verify if RNase III wt and mutants are able to dimerize, the purified proteins were incubated with increasing concentrations of DSS (disuccinimidyl suberate). Cross-linking reaction was performed in a final volume of 10 μl, including 0.5 μg of purified protein, 10 mM Hepes pH 7.4, 250 mM NaCl, 0.1 mM EDTA, 0.1 mM DTT (dithiothreitol) and increasing concentrations of DSS (1–20 μg). The reaction was performed at room temperature (~25°C) for 30 min and quenched by adding 1 μl of Tris–HCl 1 M pH 7.5 and SDS loading buffer. The samples were boiled during 5 min and then analysed in a SDS/12% PAGE gel.

### *In vitro* transcription

A canonical substrate for RNase III, called R1.1, was constructed using a synthetic DNA template and a promoter oligonucleotide obtained by commercial source (StabVida) for *in vitro* transcription, using the method described by Milligan et al. [24]. Briefly, the DNA synthetic template (0.5 μM) and the promoter oligonucleotide (0.6 μM) were annealed in 10 mM of Tris–HCl pH 8.0 by heating for 5 min at 70°C, following by incubation for 30 min, at 37°C. *In vitro* transcription was carried out using ‘Riboprobe *in vitro* Transcription System’ (Promega) and T7 RNA polymerase with a molar excess of [^32^P]-α-UTP over non-radioactive UTP. In order to remove DNA template, 1 unit of DNase (Promega) was added and incubated 30 min at 37°C. R1.1 transcript was purified by electrophoresis on an 8.3 M urea/10% PAGE. The gel slice was crushed and the RNA was eluted overnight at room temperature with elution buffer [3 M ammonium acetate pH 5.2, 1 mM EDTA, 2.5% (v/v) phenol pH 4.3]. The RNA was ethanol precipitated and resuspended in RNase-free water. The yield of the labelled substrates [cpm (counts/min)/μl] was determined by scintillation counting.

### RNase III activity assays

The activity assays were performed in a final volume of 50 μl containing the RNase III activity buffer (30 mM Tris–HCl pH 8, 160 mM NaCl, 10 mM MgCl_2_ or MnCl_2_, 0.1 mM DTT) and 10000 cpm of substrate. As a control, prior to the beginning of each assay an aliquot was taken and was incubated until the end of the assay (without the enzyme). The reactions were started by the addition of the enzyme, and further incubated at the indicated temperature. Aliquots of 5 μl were withdrawn at the time-points indicated in the respective figures, and the reactions were stopped by the addition of formamide containing dye supplemented with 10 mM EDTA. RNase activity assays were carried out at various pH values (pH 5.2, 5.4, 6.1 and 7.3), at different temperatures (4, 30, 37 and 42°C), and at different Mg^2+^ and Mn^2+^ concentrations (0.1, 1, 10, 50 and 100 mM). Reaction products were resolved in a 7 M urea/15% PAGE as indicated in the respective figure legends. Signals were visualized by PhosphorImaging and analysed using ImageQuant software (Molecular Dynamics).

### Analysis of rRNA patterns on agarose gels

*E. coli* MG1693 (wt) and SK7622 (Δ*rnc*) alone or carrying the plasmids pGEMT and pGEMT:*rnc* were grown at 37°C in LB medium to an optical density of 0.5 at 600 nm. Cells were pelleted and stored at −80°C. Total RNA was extracted following the protocol described in Stead et al. [25]. RNA was quantified on a Nanodrop 1000 machine (NanoDrop Technologies), and 15 μg of total RNA were visualized in a 1.5% (w/v) agarose gel.

### Western blot

*E. coli* MG1693 (wt) and SK7622 (Δ*rnc*) alone or carrying the plasmids pGEMT and pGEMT:*rnc* were grown at 37°C in LB medium to an optical density of 0.5 at 600 nm. Cells were pelleted and lysed by addition of 200 μl of Bug Buster reagent (Merck Millipore). After 10 min of centrifugation at maximum speed, the soluble fraction was transferred to a new tube and protein concentration was measured. 30 ng of soluble protein were separated by SDS–PAGE and transferred to a nitrocellulose membrane (Hybond ECL, GE Healthcare) by electroblotting using the Trans-Blot SD semidry electrophoretic system (Bio-Rad). Membranes were probed with a 1:10000 dilution of anti-PNPase or anti-RNase R antibodies. ECL anti-rabbit IgG-conjugated horseradish peroxidase was used as the secondary reagent in a 1:10000 dilution. Immunodetection was conducted via a chemiluminescence reaction using Amersham ECL Western Blotting Detection Reagents (GE Healthcare).

### Sequence alignment and construction of the phylogenetic tree

RNase III sequences of *E. coli, Salmonella enterica, Pseudomonas aeruginosa, Coxiella burnetii, Rhodobacter capsulatus, Lactococcus lactis, Staphylococcus aureus, Streptomyces coelicolor, C. jejuni* and *Helicobacter pylori* were aligned using Clustal W2 (http://www.ebi.ac.uk/Tools/msa/clustalw2/) [26]. The phylogenetic tree was built using Phylogeny.fr (http://www.phylogeny.fr) [27].

## RESULTS AND DISCUSSION

### Analysis of *C. jejuni* RNase III features

RNase III is a specific dsRNA endoribonuclease widely conserved in prokaryotes and eukaryotes. The amino acid alignment of the putative *Cj*-RNase III with nine different RNase III orthologues from Gram-negative and Gram-positive microorganisms reveals high conservation (Figure 1A). Indeed, *Cj*-RNase III exhibits the highly conserved amino acid stretch (NERLEFLGDS) in the catalytic domain like in all RNase III members already documented (Figures 1A and 1C) [3]. Furthermore, in Figure 1(B), the phylogenetic tree demonstrates that the sequences can be divided into two groups, which reflects well the phylogenetic relationships of RNase III between the corresponding bacteria. *Cj*-RNase III is located in a different group than *E. coli* and *S. enterica* RNase III. Among the nine microorganisms analysed, the highest similarity is observed with the *H. pylori* counterpart (48% identity).

### *Cj*-RNase III is a dimeric protein

In order to characterize the activity of *Cj*-RNase III, we cloned the *rnc* gene in an expression vector and overexpressed the protein in an *E. coli* BL21(DE3) *rec*A*rnc*105 strain. *Ec-*RNase III is able to regulate its own synthesis by cleaving near the 5′end of its own message [28]. The *rnc*105 mutation changes the highly conserved Gly^44^ residue in the catalytic domain to aspartic acid abolishing RNase III activity, whereas the *rec*A inactivation increases the stability of the recombinant plasmid. Thus, the *E. coli* BL21(DE3) *rec*A*rnc*105 strain allowed us to have higher levels of *Cj*-RNase III expression when compared with the wt strain, by preventing the cleavage of the overexpressed RNase III.

RNase III family members function as a homodimer [29]. To examine whether *Cj*-RNase III acts like a homodimer we performed a cross-linking assay using the cross-linker DSS. The protein was incubated with different amounts of DSS and the reaction was observed in a SDS/12% PAGE. DSS reacts with primary amino groups forming stable amide bonds. This will ‘fix’ the protein interactions, allowing their identification. In Figure 1(D) is possible to see the formation of a DSS-dependent specie which has two times the size of the monomeric form (~50 kDa). The result is consistent with the formation of a dimer, which led us to conclude that *Cj*-RNase III also undergoes dimerization.

### Functional complementation of *Cj*-RNase III in *E. coli*

**Figure 2:**
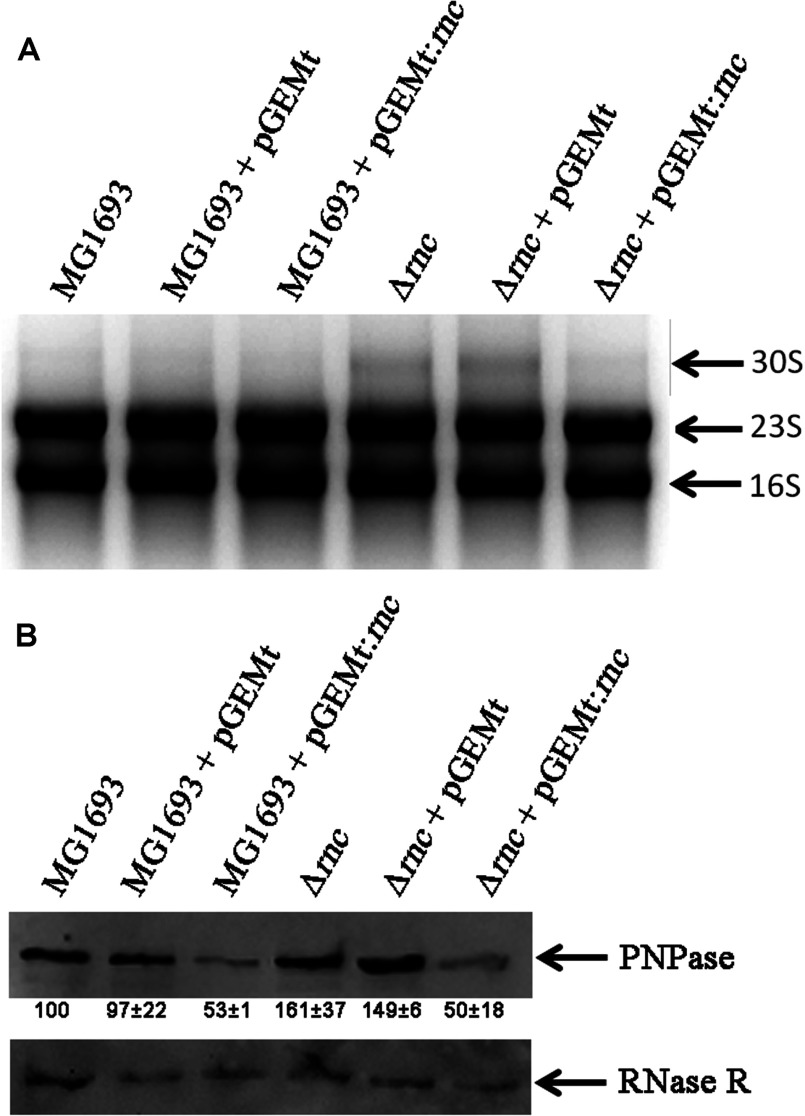
*E. coli* complementation by *Cj*-RNase III (**A**) Processing of 30S rRNA. Total RNA was extracted from *E. coli* MG1693 (wt) and SK7622 (Δ*rnc*) empty or containing pGEMt and pGEMt:*rnc* plasmids, and analysed in a 1.5% agarose gel. Bands were visualized by staining with ethidium bromide. The RNA species are indicated in the figure. This experiment was performed in quadruplicate. (**B**) PNPase regulation. Total soluble proteins were extracted from *E. coli* MG1693 (wt) and SK7622 (Δ*rnc*) empty or containing pGEMt and pGEMt:*rnc* plasmids and 30 ng of protein were applied in a SDS/Page gel. A Western blot was performed using anti-PNPase and anti-RNase R antibodies. This experiment was performed in quadruplicate. PNPase levels were quantified and the values are indicated in the figure.

RNase III is involved in the processing of 30S rRNA. In the *E. coli* strain SK7622 that has a deletion in the *rnc* gene, is possible to detect the unprocessed 30S rRNA due to RNase III absence [30] (Figure 2A). To see whether the *Cj*-RNase III was able to complement the loss of endogenous RNase III in *E. coli*, we have transformed the SK7622 (Δ*rnc*) strain with the plasmid pGEMT:*rnc* that carries the *rnc* gene from *C. jejuni* and analysed the rRNA pattern. SK7622 does not express T7 RNA polymerase, and the expression of the *Cj*-RNase III is this strain is derived from the weak expression of the plasmid. The results obtained revealed that in the presence of pGEMT:*rnc* the 30S rRNA species is no longer observed (Figure 2A). We can then conclude that *Cj*-RNase III is able to functionally replace *E. coli* RNase III in the 30S processing and that this may be also its role in *C. jejuni*.

It is also known that RNase III is involved in the posttranscriptional control of *pnp* message, which codes for the exoribonuclease PNPase. Strains deficient in RNase III present higher levels of PNPase when compared with the wt [31,32]. Since we showed that *Cj*-RNase III is able to complement an *E. coli* mutant at the level of 30S processing, we checked if the same is valid for PNPase regulation. For that purpose, we extracted the total soluble proteins and performed a Western blot using anti-PNPase antibodies. By comparing the *E. coli* parental and mutant strains, we can see that the PNPase levels are ~1.5-fold higher in Δ*rnc* and Δ*rnc*+pGEMt strains (Figure 2B). The presence of pGEMT:*rnc* plasmid that harbors *Cj*-RNase III in both parental and mutant strains, causes a substantial decrease in PNPase levels due to the presence of higher levels of this protein (Figure 2B). As a control, we used antibodies against another ribonuclease, RNase R, which regulation does not involve RNase III. We can see that for RNase R there are no changes at the protein level (Figure 2B), indicating that the modifications observed for PNPase are specific.

These complementation assays indicate that *Cj*-RNase III is efficient in the processing of *E. coli* rRNA and in the cleavage of *pnp* message, demonstrating that this enzyme is able to substitute its *E. coli* counterpart in rRNA processing and in the control of mRNA levels. This is an interesting result considering the phylogenetic distance between RNase III from *C. jejuni* and *E. coli* (Figure 1B). Similar results were previously obtained for the *Lactococcus lactis* RNase III [33], strengthening the high conservation of this family of enzymes.

### Divalent metal ion dependence of *Cj*-RNase III catalytic activity

**Figure 3:**
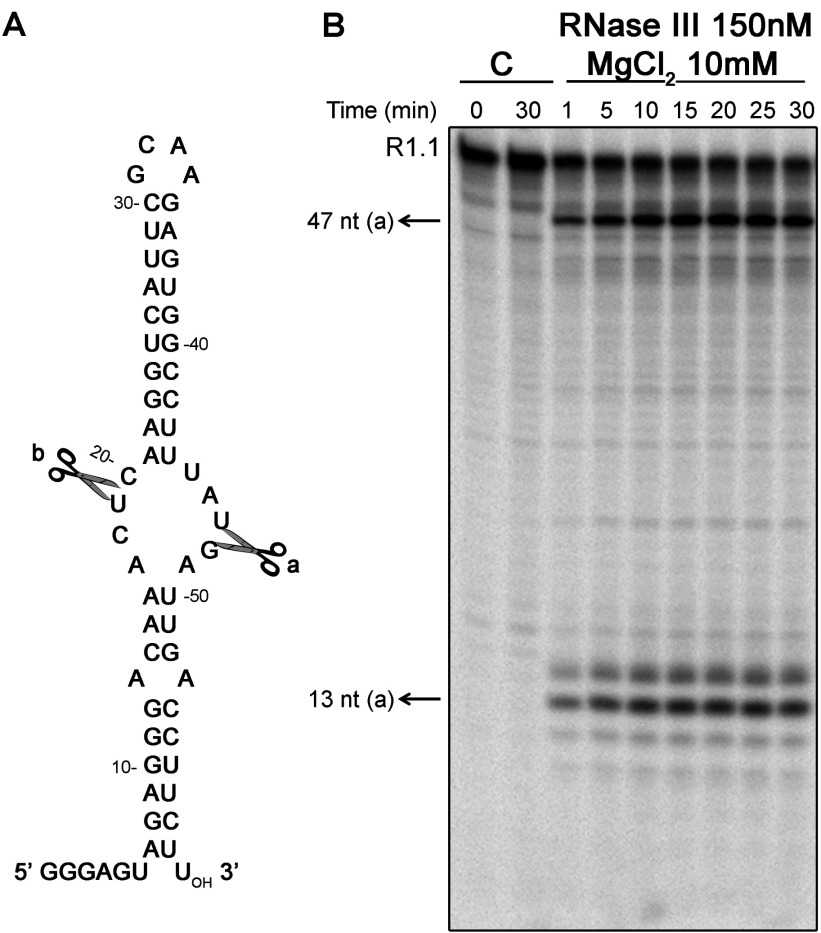
Substrate cleavage *in vitro* by *Cj*-RNase III (**A**) Sequence and secondary structure of R1.1 RNA substrate. The cleavage sites are indicated. (**B**) *In vitro* cleavage of R1.1 RNA by RNase III from *C. jejuni*. The activity assay was performed at 37°C as described in the Experimental section using Mg^2+^ as co-factor. Protein concentration is indicated in the figure. Samples were taken during the reaction at the time points indicated and reaction products were analysed in a 15% PAGE/7 M urea gel. Control reactions with no enzyme added were incubated at the maximum reaction time. Length of substrates and degradation products are indicated. These experiments were performed at least in triplicate.

R1.1 RNA is a well-known 60 nt (nucleotides) substrate for *E. coli* RNase III, which contains a single primary cleavage site (Figure 3A, (a)) that is recognized *in vivo* and *in vitro*, and a secondary site [Figure 3A, (b)] that is cleaved only under specific conditions [34]. This canonical substrate was used to check the activity of *Cj*-RNase III purified in this work. It is known that the cleavage of dsRNA by RNase III is dependent of divalent cations [7]. *Ec*-RNase III can use different divalent ions with Mg^2+^ as the preferred co-factor [35]. A cleavage assay using Mg^2+^ is shown in Figure 3(B), which reveals that *Cj*-RNase III is catalytically active and cleaves R1.1.

**Figure 4:**
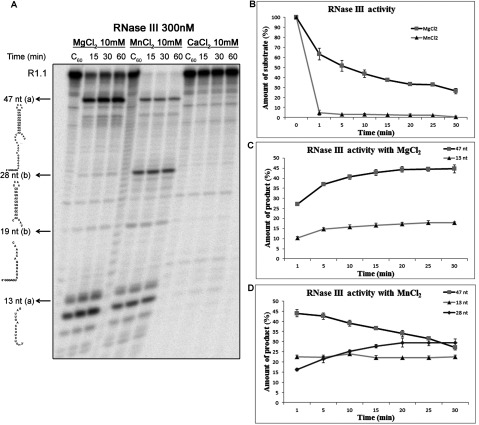
*Cj*-RNase III activity with different divalent ions (**A**) 300 nM of recombinant protein were incubated with 10000 cpm of R1.1 substrate at 37°C in a reaction buffer with MgCl_2_, MnCl_2_ and CaCl_2_. This experiment was performed at least in triplicate. (**B**) Determination of the amount of substrate degraded in the presence of 300 nM of *Cj*-RNase III with MgCl_2_ and MnCl_2_. (**C**) Determination of the amount of degradation products (47 and 13 nt) in the presence of MgCl_2_. (**D**) Determination of the amount of degradation products (47, 28 and 13 nt) in the presence of MnCl_2_.

We also tested the activity of *Cj*-RNase III in the presence of other two divalent ions, Mn^2+^ (manganese) and Ca^2+^ (calcium). It was described that Mn^2+^ is also able to support catalysis, while Ca^2+^ functions as an inhibitor [29,35]. As expected, Ca^2+^ at 10 mM does not support *Cj*-RNase III catalysis, but Mn^2+^ does (Figure 4A). However, the RNA cleavage pattern is different according to the divalent ion used. With Mg^2+^, RNase III cleaves R1.1 into two major products (47 and 13 nt) resulting from cleavage in the primary site (a) (Figure 3A), and a fraction of the substrate is not digested (Figures 3 and 4). When higher amount of enzyme is used (1200 nM) and the reaction occurs for longer periods of time (2 h), we can observe that cleavage at the secondary site (b) can also occur (results not shown). However, in the presence of 10 mM of Mn^2+^ it is possible to observe immediately the formation of the four degradation products (47, 28, 19 and 13 nt), which results from cleavage at both sites (a and b) (Figure 4A).

We have quantified the amount of substrate present at each time point of the reaction with Mg^2+^ and Mn^2+^ at the same concentration and using the same amount of protein. The graphic in Figure 4(B) shows that, after 1 min of reaction, *Cj*-RNase III degraded>95% of the substrate in the presence of 10 mM of Mn^2+^, while only ~35% is degraded in the presence of 10 mM of Mg^2+^. Moreover, in those conditions, the degradation obtained in the presence of Mg^2+^ did not reach more than 70% during the 30 min of reaction (Figure 4(B)). We also quantified the degradation products of the reaction in both conditions. In the presence of Mg^2+^, we can observe the formation of the 47 and 13 nt fragments resulting of cleavage in (a) site (Figure 4(C)). In the presence of Mn^2+^, we see a decreased in the 47 nt fragment and an increased in the 28 nt, which results at cleavage at (b) site, while the 13 nt remains constant (Figure 4D). These results support the conclusion that cleavage at (b) site is only promoted in the presence of Mn^2+^.

**Figure 5:**
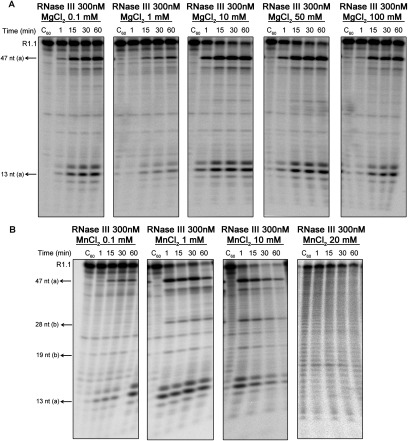
*Cj*-RNase III activity with different concentrations of divalent ions 300 nM of recombinant protein were incubated with 10000 cpm of R1.1 substrate at 37°C in a reaction buffer with different MgCl_2_ (**A**) or MnCl_2_ (**B**) concentrations, ranging from 0.1 to 100 mM. These experiments were performed at least in triplicate.

In *E. coli*, it is known that Mn^2+^ can support catalysis when used in a concentration range of 0.1–1 mM. For higher concentrations (> 5mM), Mn^2+^ was shown to have an inhibitory effect [35]. In addition, *Ec*-RNase III cleaves R1.1 substrate with the same efficiency at the optimal concentrations of Mg^2+^ and Mn^2+^ [36]. Thus, we decided to test the activity of *Cj*-RNase III in a broad range of Mg^2+^ and Mn^2+^ concentrations from 0.1 to 100 mM. The results presented in Figure 5 indicate that this protein is active over a wide range of Mg^2+^ concentrations. However, it seems to prefer values of the order of 10–50 mM (Figure 5A) since we can observe higher consumption of the substrate. Moreover, in the presence of Mn^2+^ the protein is most active between 1 and 10 mM (Figure 5B). When higher concentrations are used (above 20 mM), the reaction is inhibited. Overall, our experiments show that in contrary to what was reported for *E. coli* RNase III, the Mn^2+^ seems to be the preferred co-factor of *Cj*-RNase III. Also, Mn^2+^ shows to have an inhibitory effect when increased concentrations are used, however the enzyme supports a higher concentration of this metal ion when compared with *E. coli* counterpart.

Our study strengthen the idea that Mn^2+^ and Mg^2+^ ions are responsible to control or adapt ribonuclease activity. Very interestingly, a recent study suggests that RNase HII from *Pyrococcus furiosus* digests dsRNA in the presence of Mn^2+^ ions, whereas there is no dsRNA digestion activity using Mg^2+^ in the reaction [37].

The maintenance of metal homoeostasis is essential for proper functioning of the cell, and has significant implications for microbial adaptations in changing environments. Bacteria have evolved several mechanisms to acquire metals and to sustain growth in environments with limited amounts of free trace metals [38]. Manganese is an example of a cofactor that could replace the role of Mg^2+^ in certain proteins that has emerged as a very important metal in virulence [39]. It is known that macrophages have a poor magnesium, acidic and low oxygen environment [39]. Curiously, divalent manganese is thermodynamically favored in acidic environments and in the absence of oxygen. *Campylobacter* is able to survive within macrophages for a period of 24–30 h. It is within the macrophages that Mn^2+^ homoeostasis is thought to be important for *C. jejuni*; however, the specific cellular role of this metal is not yet established [40]. It raises the question if a possible shift of the metal cofactor could be related with an adaptation of the enzyme activities to new conditions. The results obtained in this work lead us to speculate that *Cj*-RNase III may have an important role under a Mn^2+^-rich environment, namely during invasion and infection.

### pH dependence and thermostability of *Cj*-RNase III catalytic activity

**Figure 6:**
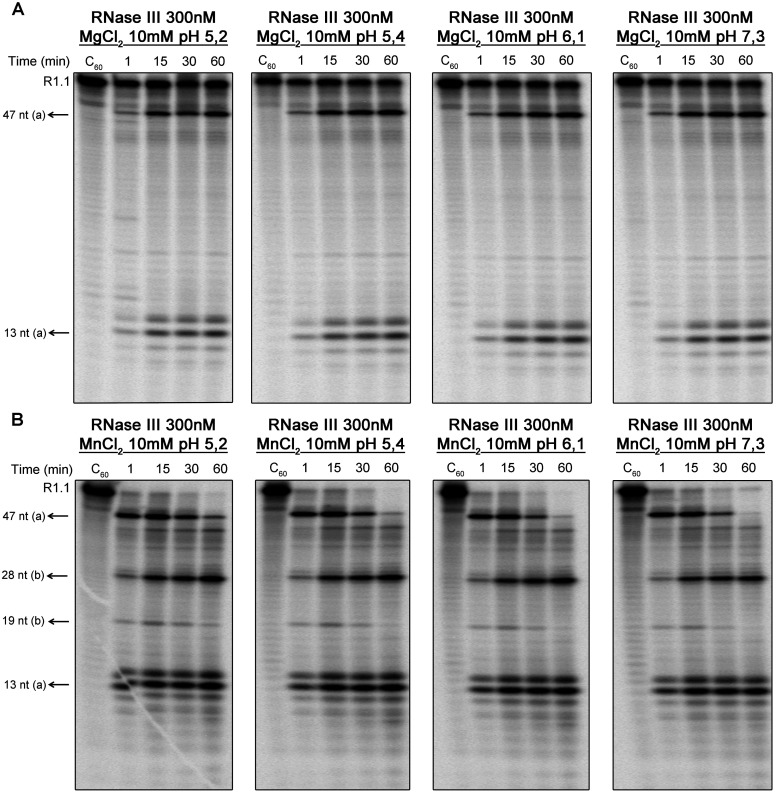
*Cj*-RNase III activity using different pH values 300 nM of recombinant protein were incubated with 10000 cpm of R1.1 substrate at 37°C in a reaction buffer with MgCl_2_ (**A**) or MnCl_2_ (**B**) and different pH, ranging from 5.2 to 7.3. These experiments were performed at least in triplicate.

*C. jejuni* faces different pH values during the infection process, and is able to survive during the passage from food or water source into the host gastrointestinal tract. Thus, this pathogen is prepared to sense and respond to a rapid decrease of pH (normally below the threshold for growth) [41]. It is known that the pH of a human stomach depends on physiological variables. Following transit through the stomach, bacteria encounter a variety of environments within the intestine, ranging from mildly acidic (pH 5.5) to moderately alkaline (pH 7.4) [42,43]. Taking this into account, we have tested the activity of *Cj*-RNase III at different pH values between 5.2 and 7.3, in the presence of 10 mM Mg^2+^ or Mn^2+^. As showed in Figure 6, *Cj*-RNase III is active in all pH tested, and it seems that the protein was efficient in cleaving R1.1 RNA even in the mildly acidic comparing with the standard conditions (Figure 3).

**Figure 7:**
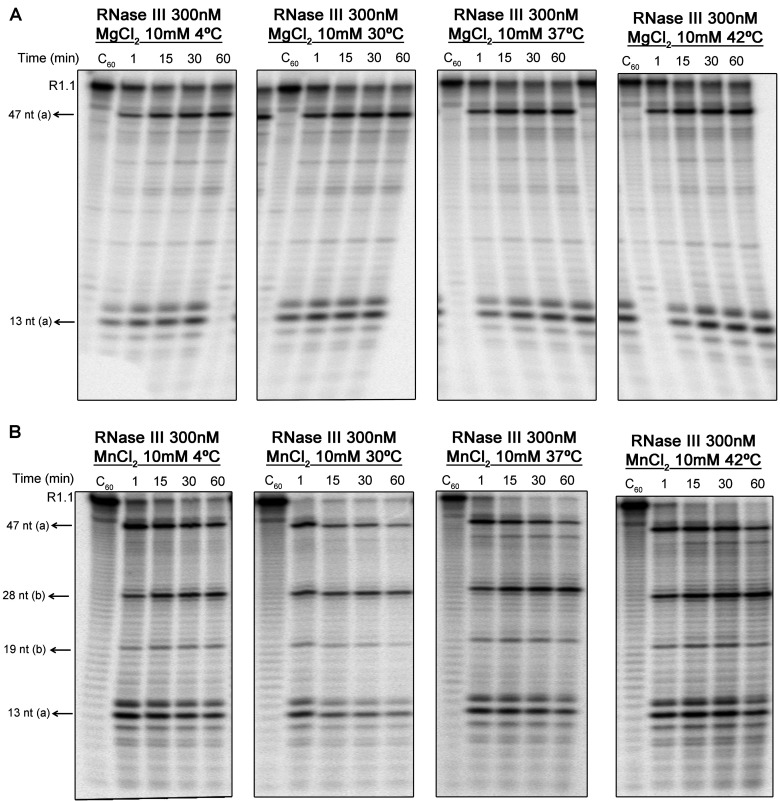
*Cj*-RNase III activity at different temperatures 300 nM of recombinant protein were incubated with 10000 cpm of R1.1 substrate at 4, 30, 37 and 42°C in a reaction buffer with MgCl_2_ (**A**) or MnCl_2_ (**B**). These experiments were performed at least in triplicate.

Differences of temperature constitute another key factor during *C. jejuni* survival. Although this microorganism is able to grow at 37°C, its optimal temperature is about 42°C, the avian body temperature [44]. Moreover, it has the potential to survive under refrigerated temperatures [45]. The ability of *C. jejuni* to survive refrigeration is of major interest to food safety and public health, since it is a typically intervention used in the control of bacterial growth in food. For this reason, we decided to analyse the activity of *Cj*-RNase III at different temperatures: 4, 30, 37 and 42°C. The results obtained show that *Cj*-RNase III is active in an unexpectedly large range of temperatures from 4 to 42°C (Figure 7).

Altogether, these experiments showed that *Cj*-RNase III is active in a wide range of conditions. The pH tolerance of *Cj*-RNase III and its capability to cleave over different temperatures, including 4°C could be indicative of a possible role of RNase III during the acid shock and temperature response of *Campylobacter* during the infection process.

### Analysis of the effect of point mutations in the activity of *Cj*-RNase III in the presence of Mg^2+^ and Mn^2+^

Structural advances combined with directed mutagenesis provide powerful tools to uncover important issues of RNase III catalytic activity. RNase III orthologues have eight highly conserved aspartate (D) and glutamate (E) residues in the catalytic domain (E38, E41, D45, E65, E100, D114, E117 and D126 in *E. coli*), which were suggested to have an important role in activity. In *E. coli* the metal binding is coordinated by four of these residues: E41 and D45 that belong to the signature box (NERLEFLGDS), and D114 and E117 [5]. Substitution of D45 for an alanine (*Ec*-D45A) had a severe impact on the catalytic function of *Ec*-RNase III both *in vitro* [46] and *in vivo* [5]. The replacement of E117 by an alanine or lysine also blocked the phosphodiester hydrolysis, and was also reported to suppress RNase III activity *in vivo* [29]. Additionally, two other substitutions of E117 residue were performed, in which this amino acid was substituted for an aspartic acid (*Ec*-E117D) and for a glutamine (*Ec*-E117Q) [36]. The introduction of these mutations in *Ec*-RNase III induced minimal structural changes but affected the activity of the enzyme in the presence of Mg^2+^. Curiously, the presence of Mn^2+^ in the reaction rescued the activity of the *Ec*-D45A and *Ec*-E117D mutant [36,46]. Considering the importance that Mn^2+^ plays in the activity of *Cj*-RNase III, we decided to alter the equivalent E117 in *C. jejuni* protein (*Cj*-E116) for aspartic acid and glutamine and see the effect of these mutations in the activity of the enzyme. We also substituted the equivalent residue D45 (*Cj*-D44) for an alanine and checked its effect on catalysis.

**Figure 8:**
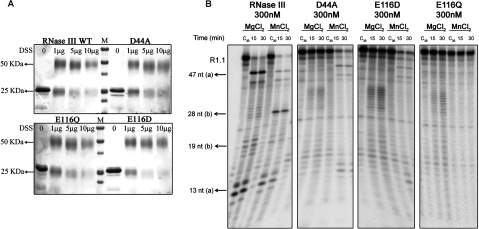
Characterization of *Cj*-RNase III mutants (**A**) Cross-linking of RNase III mutants from *C. jejuni* using DSS. Roughly 0.5 μg of protein was incubated with increasing concentrations of DSS as indicated in the figure. Proteins were visualized by Coomassie Brilliant Blue staining. (**B**) *Cj*-RNase III mutants activity in the presence of Mg^2+^ and Mn^2+^. Roughly 300 nM of protein were incubated with 10000 cpm of R1.1 substrate at 37°C in a reaction buffer with MgCl_2_ or MnCl_2_. These experiments were performed at least in triplicate.

As already mentioned, in order to be active, the nuclease domains of RNase III undergoes dimerization. After purification of the mutant proteins, their ability to form dimers was tested by incubation the DSS cross-linker. The mutants exhibited normal homodimeric behavior, comparable with the wt protein (Figure 8A).

We also analysed the capacity of the mutant proteins to support catalysis. For this purpose, we tested the activity of the enzymes in the conditions where the wt *Cj*-RNase III carries out efficient degradation of the RNA substrate, in the presence of Mg^2+^ or Mn^2+^. Using Mg^2+^ as cofactor, the results reveal that the mutants do not cleave efficiently R1.1 RNA (Figure 8B). However, for the three mutants, it is possible to observe a residual activity. The same was verified for the equivalent mutants in *E. coli* when extended reaction times and high enzyme concentrations were used [36,46]. These data suggest a conservation of the role of these residues in the catalytic mechanism of action of RNase III.

Regarding the use of Mn^2+^ in the reactions, we have shown that this metal ion was able to rescue the activity of *Cj*-D44A and *Cj*-E116D mutants. As previously discussed, in the presence of Mn^2+^, the wt *Cj*-RNase III is able to recognize the secondary site (b) of R1.1 RNA (Figures 3 and 4). Nevertheless, *Cj*-D44A and *Cj*-E116D mutants are no longer able to cleave at this site (Figure 8b), even when higher amounts of protein are used (results not shown). When dsRNA is cleaved, two hydrolysis events occur at each active center of RNase III. Moreover, it is known that the residues D44 and E110 in *A. aeolicus* (D45 and E116 in *C. jejuni*, respectively) are responsible for a second RNA-cutting site [5]. This is in good agreement with the lost of *Cj*-D44A and *Cj*-E116D mutants to recognize and cleave the R1.1 substrate at the secondary site.

Regarding the *Cj*-E116Q mutant, we demonstrated that Mn^2+^ was not able to restore its catalytic activity (Figure 8B). Our findings are consistent with the weak affinity of the carboxamide group for divalent metal ions as previously discussed by Sun and Nicholson, which explains the inability of Mg^2+^ and Mn^2+^ ions to restore the activity of this mutant [36].

Overall, these results show that D44 and E116 residues are important for the catalytic activity of *Cj*-RNase III by providing a metal-binding site, which is recognized by Mg^2+^ and Mn^2+^. The mutants are not able to cleave in the presence of Mg^2+^, but a different behaviour is seen in the presence of Mn^2+^. Moreover, our data strengthen that in fact RNase III family of enzymes contains a conserved mechanism of action.

In this paper, we have undertaken a functional and biochemical analysis of *Cj*-RNase III. We demonstrated that *Cj*-RNase III is able to complement an *E. coli rnc* deficient strain in 30S rRNA processing and in regulation of PNPase levels. These observations reinforce the conservation of these functions in bacteria, since similar results were previously obtained for other microorganisms [33]. We were also able to show that *Cj*-RNase III is active in a wide range of conditions. Such plasticity may be important for the adaptation of this pathogen to different conditions during infection. The data obtained lead us to speculate that *Cj*-RNase III may have an important role under a Mn^2+^-rich environment, contrarily to the consensus that Mg^2+^ is the physiologic relevant cofactor for RNase III orthologues.

PNPase has an impact on cell biology of *C. jejuni*, namely in survival at refrigerated conditions, bacterial swimming, colonization, cell adhesion and invasion. Moreover, its levels are controlled in an RNase III-dependent manner. Taking these into account, RNase III might be also a main player in the adaptation of this pathogen to environmental demands. Hence, it is crucial to better understand the mechanism of action of this endoribonuclease.
